# Correlation between self-efficacy, fear of movement, empowerment, enablement, and number of visits to physiotherapist among patients with musculoskeletal disorders in primary health care: a feasibility study

**DOI:** 10.1186/s40814-022-01101-4

**Published:** 2022-07-06

**Authors:** Madelene Törnblom, Eva Ekvall Hansson

**Affiliations:** 1 Åparkens Vårdcentral, SE- 28232 Tyringe, Sweden; 2 Department of Health Sciences, Faculty of Medicine, Lund University, SE-22100 Lund, Sweden

**Keywords:** Primary health care, Physical therapists, Musculoskeletal diseases, Self-efficacy, Feasibility studies

## Abstract

**Background:**

Musculoskeletal disorders are a costly burden for health care and social care services. Patients with musculoskeletal disorders are often treated by physiotherapists in primary health care. Psychosocial variables can be a significant obstacle to recovering from musculoskeletal injuries.

The primary aim of this pilot study was to assess the feasibility of performing a prospective study investigating whether self-efficacy, fear of movement, empowerment, or enablement has any relation to the number of visits to physiotherapists among patients with a musculoskeletal disorder in primary health care.

**Methods:**

Prospective study with a consecutive selection including eleven female and eight male patients age ranged between 22 and 82 years old seeking physiotherapist for the first time for a musculoskeletal disorder in primary health care. Primary outcome measures included operational and practical feasibility regarding recruitment of participants, use of questionnaires, and key variables to be collected as part of the study. Secondary outcomes included the correlation between self-efficacy (Exercise Self-Efficacy Scale (ESES-S)), fear of movement (Tampa Scale for Kinesiophobia (TSK-SV)), empowerment (Making Decisions Scale), enablement (Patient Enablement Instrument (PEI)), and the number of visits to physiotherapists. Statistical analysis was done using IBM SPSS statistics version 28 with analysis of correlation using Spearman’s rank correlation coefficient.

**Results:**

Nineteen patients accepted to participate in the study and were included in the final analysis. Between 14 and 18 completed questionnaires were included.

There was a statistically significant correlation between the number of visits to the physiotherapist and self-efficacy, rho=0.692 and *p*=0.006.

**Conclusion:**

The results of the study showed that the design is feasible in terms of recruitment of participants and use of questionnaires. New variables to collect in a large-scale study were identified. In a large-scale study, attention needs to be focused on the improvement of the number of completed questionnaires. The results of this study indicate that the present care of patients with a low level of self-efficacy is not optimal.

## Key messages regarding the feasibility

- What uncertainties existed regarding the feasibility?

There were uncertainties regarding the questionnaires and the recruitment process.

- What are the key feasibility findings?

The inclusion and exclusion criteria were relevant and easy to use in a clinical setting. The recruitment process took 5 months, which was expected considering the size of the health care centre. Nineteen participants were included from 28 eligible patients and the recruitment rate was 68%. Fourteen of the participants completed correctly the ESES, TSK, and the Empowerment scale and eighteen completed correctly the PEI. We identified new variables to collect in a future large-scale study.

- What are the implications of the feasibility findings for the design of the main study?

Variables, such as the previous experience of physiotherapy, physical activity, sedentary behaviour, self-reported health, and visits to physiotherapists individually and in a group, will be collected in a future large-scale study.

## Background

Worldwide approximately 1.71 billion people have musculoskeletal conditions with low back pain contributing most as a leading cause of disability in 160 countries with a prevalence of 568 million people [1]. Musculoskeletal conditions can significantly limit mobility and lead to need for rehabilitation [1]. It can also contribute to early work retirement, reduced ability to participate in society, and lower levels of well-being [1]. Worldwide, population is both increasing and ageing, thus potentially increasing the number of people with musculoskeletal conditions [1]. Musculoskeletal conditions include more than 150 different diagnoses [2]. They are characterized by pain and reduced physical function and can often lead to significant mental health decline and increased risk of developing other chronic health conditions [2]. In the adult population in welfare states, musculoskeletal conditions cause more functional limitations than other groups of disorders [1].

Medically defined pain is the reason for 30% of all patients to visit a general practitioner [3]. Among these, two-thirds had pain from the musculoskeletal system, 37% of whom had acute pain and 37% chronic pain [3]. The incidence of musculoskeletal complaints in general practice in the Netherlands in 2009 was 268 patients per 1000 [4]. Dorsalgia was the fourth most common diagnosis in primary care in Sweden in 2011 affecting 2.56 % of the population [5]. The prevalence of patients seeking care due to different musculoskeletal disorders at primary health care centres in Sweden was almost 60% [6]. Primary health care is the most common care provider for patients with acute and chronic pain [7]. For patients in primary care with musculoskeletal disorders, the physiotherapist can be considered as the primary assessor [8] and is more cost-effective than the first visit to a general practitioner [9].

About half of the patients visiting physical therapy practice suffer from back pain, neck complaints, shoulder complains, and knee complaints. The duration of complaints can vary, but the most common duration is less than 1 month. One-third of the patients have recurrent complaints and a majority of the patients have previously been to physical therapy [4].

For patients in physical therapy in primary care, the median number of treatment sessions is seven for referred patients and six for self-referred patients [4]. Self-referred patients have on average three sessions less than referred patients when standardized for diagnosis, age, and sex [4]. In on-site clinical settings and physiotherapy practice studies have shown a mean number of visits between 5.7 and 9.6 and that number of visits can vary by treated body area [10, 11].

Fifty percent of the patients visiting primary care for nonspecific back or neck pain can be expected to report pain and disability after 5 years [12]. Identifying subgroups of patients with a better or poorer outcome over time can help in selecting the most suitable treatment programme [13]. Sullivan and Adams suggest that it may become possible for physiotherapists to detect and intervene on risk factors for prolonged pain and disability at the primary care level and prevent the development of chronicity [14].

Psychosocial variables can be a significant obstacle to recovering from musculoskeletal injuries [15]. In the context of health research, psychological factors can be seen as mediating the effects of social structural factors on individual health outcomes [16]. Psychosocial risk factors may include emotional reactions such as fear, relational factors such as conflict and lack of support, and predisposing factors such as attitudes and beliefs [17]. The presence of these factors indicates that pain-related disability will persist [17]. Kinesiophobia, self-efficacy, empowerment, and enablement can be included in psychosocial variables.

Kinesiophobia describes an excessive, debilitating, and irrational fear of physical movement emerging from a feeling of vulnerability for painful re/-injury [18]. Fear of movement/(re)injury explained variance in pain-related disability pre-treatment [19]. A review from 2018 found strong evidence for an association between a greater degree of kinesiophobia and greater levels of pain intensity and disability [20]. Moderate evidence was found that a greater degree of kinesiophobia predicts the progression of disability over time [20].

Perceived self-efficacy is described as a person’s beliefs in his or her ability to successfully execute the required behaviours in specific situations or tasks to accomplish the desired outcomes [21]. The sense of self-efficacy can play a large role in how the person deals with challenges [21]. Gatchel et al. state that high self-efficacy is beneficial when people are confronted with acute or chronic pain [15]. People with high self-efficacy are likely to engage in health-promoting behaviours because of higher expectations of performance success and they are more likely to continue with health-promoting behaviours even when they face obstacles [15]. Self-efficacy was the most salient predictor of pain-related disability for patients with musculoskeletal pain consulting a physiotherapist in primary care [19].

A meta-analysis from 2014 investigated the relationship between self-efficacy and pain severity, functional impairment, and affective distress in chronic pain samples [22]. Self-efficacy had negative overall correlations with pain severity, impairment, and affective distress [22]. The authors stated that self-efficacy is correlated to main outcomes for patients with chronic pain and that high self-efficacy is also a potentially important protective factor and low self-efficacy e potentially important risk factor [22].

Strong evidence that self-efficacy at baseline predicts outcome was found in a systematic review of randomized controlled trials investigating self-management for persons with chronic musculoskeletal pain [23]. The systematic review also found strong evidence that physical activity and pain catastrophizing can mediate outcomes from self-management [23]. The development of interventions that could early detect and treat psychosocial risk factors for poor recovery from musculoskeletal disorders is desirable [17].

Empowerment describes the individual’s determination over one’s own life and democratic participation in the community [24]. It is a psychological sense of personal control or influence [24]. Studies about the concept of empowerment as a predictor of outcome in patients with musculoskeletal disorders seem to be lacking. In patients with fibromyalgia, psychological empowerment is a relevant factor correlated with health outcomes [25]. In the field of diabetes care, empowerment is more frequently used and it is suggested that empowerment may help improve medical outcomes in chronic conditions [26]. McAllister et al. write that patient empowerment needs to be considered as a measurable outcome from healthcare services and that more research is needed [27].

To investigate the quality of care in clinical practice, patient satisfaction has been used as a common outcome measure, but it has been argued that satisfaction measures patient’s expectations rather than the actual outcome [28]. Enablement can be used as measuring consultant quality and is described as the patient’s experience of the consultation and the impact of the consultation on a patient’s self-perceived ability to understand and cope with their health and illness [29]. Enablement examines other aspects of a clinical consultation than patient satisfaction [29]. The relationship between the fulfilment of specific patient expectations and enablement is uncertain [30]. Brusse and Yen state that the nature of enablement is not fully investigated and more research is required before enablement is used in making decisions on policy and practice [30]. A recent study stated that PEI, after further development, can be a valid outcome measure used in the long-term management of patients with chronic musculoskeletal pain [31].

The physiotherapists’ role in the field of behavioural medicine is to help the patients become more independent regarding their health issues. This is accomplished by providing the patients with self-care programmes often including home exercise programmes and by giving the patient tools to manage relapses before they even occur [32].

For some patients, self-care programmes are not enough; they need more support from the physiotherapist such as exercising at the clinic and more frequent visits. Is it possible to detect these patients that are in need of more early support?

To the authors’ knowledge, there is a lack of studies that investigate if self-efficacy, fear of movement, empowerment, or enablement can affect the number of visits to the physiotherapist for patients with musculoskeletal disorders.

## Aim

The primary aim of this pilot study was to assess the feasibility of performing a prospective study investigating whether self-efficacy, fear of movement, empowerment, or enablement has any relation to the number of visits to physiotherapists among patients with a musculoskeletal disorder in primary health care.

## Method and material

### Study design

A prospective pilot study reported according to the CONSORT statement [33].

### Settings and sample

The selection was done consecutively between September 2017 and January 2018 in a health care centre in the southern part of Sweden. The selection continued until the set number of patients agreed to participate and completed the questionnaires. The health centre has 6800 enrolled patients, and three physiotherapists are included in the staff.

**Fig. 1:**
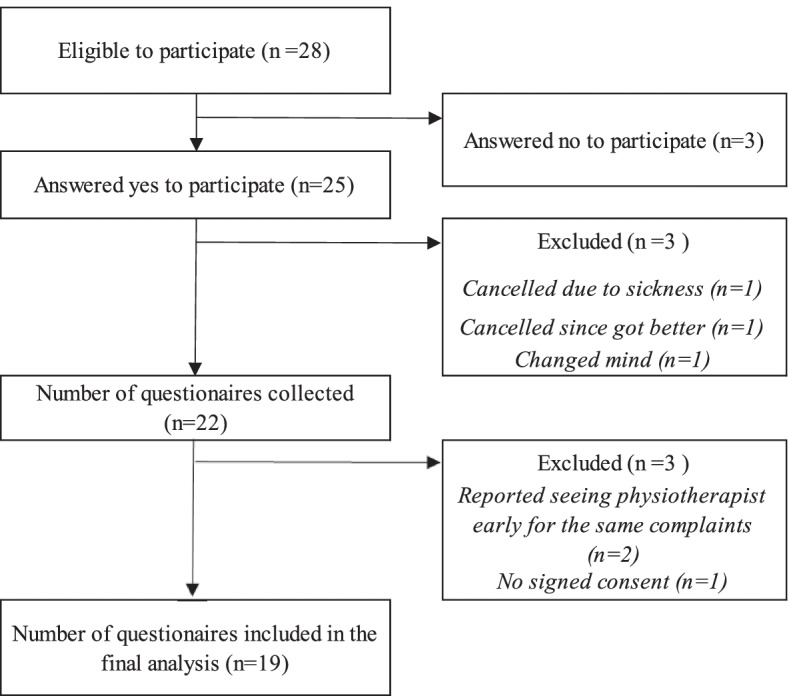
Flow chart illustrating the recruitment process

A total of 28 patients met the inclusion criteria and were asked to participate in the study (Fig. 1). Twenty-five patients answered yes. Three patients changed their minds or did not show up to their first meeting due to sickness or that they had gotten better and cancelled their appointment. A total of 22 questionnaires were collected. Three questionnaires were excluded. In one, there was no written consent, and in two, the patients reported having seen a physiotherapist earlier for the same symptom/symptoms. A total of 19 questionnaires were included in the analysis.

### Inclusion criteria

Patients, who seek physiotherapists for the first time for a musculoskeletal disorder, are over 18 years of age and can understand both spoken and written Swedish.

### Exclusion criteria

Patients who have visited physiotherapists previously for the same musculoskeletal disorder and/or are diagnosed with malignant diseases. Patients, who suffer from dementia, severe mental illness, or other conditions such that they were unable to understand the information about the study and not able to fill in the questionnaires.

### Procedure

The patients were informed by a physiotherapist about the possibility to participate in the study when they either contacted the clinic by telephone or visited the clinic in person to make an appointment because of a musculoskeletal disorder. If the patient agreed to participate, data collection was acquired through three questionnaires that patients filled in before the first visit to a physiotherapist and one after the first visit to a physiotherapist. A review of medical records data after the last visit was also performed. Information was collected about age, gender, educational level, work status, how long they have had the current symptom/symptoms, if they previously had met a physiotherapist for other disorders, and if they had previously sought a physiotherapist and/or a doctor for present symptom/symptoms. Before the first visit, the patient filled in the Swedish version of the Exercise Self-Efficacy Scale (ESES-S), the Swedish version of the Tampa Scale for Kinesiophobia (TSK-SV), and the Making Decisions Scale. After the first visit, the patient filled in the Patient Enablement Instrument (PEI). Written consent to participate in the study was collected before the patient filled in the first questionnaire and the collection of medical records was retrieved 1 year after inclusion. The questionnaire was handed out by a receptionist that registered the visit and/or the physiotherapist that saw the patient. Patients filled in their questionnaires without the attending physiotherapist present. They sealed the envelope and either put it in a box at the physiotherapist’s office or handed it to the physiotherapist.

When the contact between the physiotherapist and the patient for the specific symptom/symptoms ended, information about the number of visits to the physiotherapist as well as information about diagnosis and the results of the treatment period was gathered. The review of medical records was done a year after the selection occurred.

### Primary outcome measures

Operational and practical feasibility regarding recruitment of participants, use of questionnaires, and key variables to be collected as part of the study. More specifically, we wanted to study the recruitment process in terms of recruitment rate and time to recruit the set number of participants. We also wanted to evaluate if the inclusion and exclusion criteria, according to the physiotherapists conducting the study, were relevant and easy to use in a clinical setting. Recruitment was measured as to how many patients were included in the study in relation to eligible patients and how many questionnaires were completed correctly. We also wanted to identify if there where are any other variables that would be useful in a large-scale study and if there were any other changes to be done when performing the large-scale study.

### Secondary outcome measures

#### Exercise Self-Efficacy Scale

ESES has been translated to Swedish in ESES-S (Appendix 1) [34]. The questionnaire consists of six questions all describing common barriers to exercise [35]. In the Swedish version, the answers have a range from “not certain” (1point) to “very certain” (10 points) [34]. The total score ranges between 6 and 60 points [34]. The ESES-S has respectable internal consistency and moderate test-retest reliability in people with rheumatoid arthritis [36]. Construct validity was partially supported [36].

#### Tampa Scale for Kinesiophobia

TSK is a questionnaire created to evaluate kinesiophobia [18]. TSK has been translated to Swedish in TSK-SV (Appendix 2) and tested for reliability and validity on adult persons and patients with chronic low back pain [37]. The original TSK consists of 17 questions where the patient can “strongly disagree” to “strongly agree” to all the questions on a scale from 1 to 4 [18]. In TSK, four items are inverted (numbers 4, 8, 12, and 16) [18]. The total score for the TSK ranges from 17 to 68 [18]. A high TSK score indicates a high degree of kinesiophobia [37].

#### The Empowerment Scale-Making decisions scale

The Empowerment Scale is a scale created to measure the personal construct of empowerment [38]. Five factors of empowerment are measured in the Empowerment Scale: self-esteem, self-efficacy, power-powerlessness, community activism and autonomy, optimism and control over the future, and righteous anger [38]. The total score is a reliable and valid measure [39]. The Swedish version of the Empowerment Scale is called Making Decisions (Appendix 3) [40]. Making decisions was tested and confirmed valid for persons with mental illnesses [40]. It is a 28-item instrument in which respondents answer questions on a 4-point scale ranging from strongly agree to strongly disagree [38]. The total score ranges from 28 to 112 [38].

#### Patient Enablement Instrument

PEI is used to investigate the impact of a consultation on a patient’s self-perceived ability to understand and cope with their health and illness [29]. PEI has been translated to Swedish (Appendix 4) and tested for its reliability in a Swedish general primary health care population [41]. The Swedish version of the PEI instrument has moderate to good reliability and high internal consistency [41]. The PEI has shown fair content validity, construct validity, and internal consistency [31]. It is recommended to use research factors at the group level related to enablement [41]. PEI consists of six questions. The answers able to give are “same or less”, “not applicable” (0 points), “better/more” (1 point), and “much better/much more” (2 points) [29]. The total PEI score ranges from 0 to 12 [29].

### Ethical considerations

The study was carried out in accordance with the Declaration of Helsinki [42]. All participation in the study was voluntary, which was made clear in the oral and written information given before entering the study. The written information described which information was collected from questionnaires and medical records. The participants gave their written consent to enter the study. The therapists at the clinic were told not to ask about the content of the questionnaire and the patients were informed that their data is treated confidentially and that no individual will be able to be identified in the results. The data collected from questionnaires and medical records are stored in a locked cabinet at the author’s workplace. The study was approved 2016-01-26 by the Regional Ethical Review Board in Lund, Sweden (No. 2015/918). A risk with this study is the time it takes for the patients to fill in the questionnaires. The benefit that the results of this study can have will weigh this up.

### Statistical analysis

Power calculation revealed that with a mean number of visits set to 6, a standard deviation of 1.5, and a clinically important difference set at 0.6 visits, a sample size of in total 200 patients will give a power of 80% with the *p*-value set at 0.05. For this pilot study, a sample size of 10% of the total sample size was considered appropriate [43]. In Region Skåne, the mean number of visits to a physiotherapist is 6 (personal communication with health care officer in primary health care).

Due to the small sample size and not normal distribution of the variables, statistical analysis of the correlation between self-efficacy, fear of movement, empowerment, enablement, and the number of visits to physiotherapists was done using Spearman’s rank coefficient (non-parametric test). Descriptive statistics are presented by number (n and percentage). The analysis was done using IBM SPSS statistics version 28.

## Results

The inclusion and exclusion criteria were, according to the physiotherapists conducting the recruitment, relevant and easy to use in a clinical setting. The recruitment process took 5 months, which was expected considering the size of the health care centre. Nineteen participants were included from 28 eligible patients and the recruitment rate was 68%. Characteristics of the study sample are shown in Table 1.

**Table 1:** Demographic description of the participants in the study

Age in years, mean (SD) *n*=18	57 (18)
Sex, *n*=19	
Male, *n* (%)	8 (42)
Female, *n* (%)	11 (58)
Educational level, *n*=19	
Nine-year compulsory school, *n* (%)	5 (26)
Upper secondary school, *n* (%)	8 (42)
University, *n* (%)	6 (32)
Occupation, *n*=18	
Worker, *n* (%)	7 (39)
Student, *n* (%)	1 (57)
Retired, *n* (%)	9 (50)
Job-seeker, *n* (%)	1 (5)
Duration of symptoms, *n*=19	
Less than a week, *n* (%)	1 (5)
A week to a month, *n* (%)	2 (11)
A month to three months, *n* (%)	5 (26)
More than three months, *n* (%)	11 (58)
Previous meet doctor for current symptoms, *n*=14	
Yes, *n* (%)	6 (43)
No, *n* (%)	8 (57)
Previously met physiotherapist for other symptoms, *n*=15	
Yes, *n* (%)	15 (100)

Valid percent is shown if n<19

The median number of visits was 2 with an interquartile range (IQR) of 5 (Table 2). A total of 30 diagnoses were set, including 20 different diagnosis codes.

**Table 2:** Results of the treatment

		*N*=19
Number of visits		
Median		2
Interquartile range		5
Percentiles		
	25	2
	50	2
	75	7
Minimum		1
Maximum		28
Reason for ending the contact		
Unclear, *n* (%)		1 (5)
Failed to show up, *n* (%)		2 (11)
Cancelled, *n* (%)		1 (5)
Continued exercise programme on their own, *n* (%)		12 (63)
Has recovered, *n* (%)		2 (11)
Referred to other treatment, *n* (%)		1 (5)
Result		
Unclear, *n* (%)		3 (16)
No follow-up, *n* (%)		3 (16)
No effect of treatment, *n* (%)		1 (5)
Little better, *n* (%)		2 (11)
Better, *n* (%)		6 (32)
Much better, *n* (%)		2 (11)
Recovered, *n* (%)		2 (11)

Ten out of 19 patients (53%) got better or recovered after treatment. The reason for ending the contact with the physiotherapist was for twelve out of 19 patients (63 %) that they wanted to continue their exercise programme on their own (Table 2).

Fourteen of the participants correctly completed the ESES, TSK, and the Empowerment Scale and eighteen completed correctly the PEI (Table 3).

**Table 3:** Correlation between outcome measures and number of visits to the physiotherapist

	(*n*)	Rho (*p*)	95% confidence intervals (2-tailed)	
			Lower	Upper
ESES	14	0.692** (0.006)	0.238	0.898
TSK	14	−0.203 (0.487)	−0.672	0.382
The empowerment scale	14	0.208 (0.476)	−0.378	0.675
PEI	18	−0.133 (0.600)	−0.575	0.369

**Correlation is significant at the 0.01 level (2-tailed)

*ESES* Exercise Self-Efficacy Scale, *TSK* Tamps Scale for Kinesiophobia, *PEI* Patient Enablement Instrument

There was a statistically significant correlation between the number of visits to the physiotherapist and self-efficacy rho=0.692 and *p*=0.006 (Table 3). The 95% confidence interval was 0.238 to 0.898 (Table 3). No other correlation between the number of visits to the physiotherapist and fear of movement, empowerment, or enablement was statistically significant (Table 3).

## Discussion

In this feasibility study, the recruitment rate was high at 68%. The number of completed questionnaires was considered acceptable, but able to be improved. We identified possible improvements of the design before conducting a large-scale study, regarding the procedure as well as questionnaires and variables to collect. There was a statistically significant correlation between the number of visits to the physiotherapist and self-efficacy. No other statistically significant correlation was found. The median number of visits was 2 with an IQR of 5 and a range between 1-28.

The use of questionnaires is commonly used to address this kind of research question. The questionnaires were handed out and completed in the clinic, which is relevant. For all 19 participants, there was information about the number of visits, diagnosis, reasons for ending the contact, and results of the treatment. Fourteen of the participants correctly completed the ESES, TSK, and the Empowerment Scale and eighteen correctly completed the PEI (Table 3). Some of the participants missed out on filling in the reverse side of the papers. Clearer instructions and a reminder of this could have improved the answer rates.

There was a statistically significant correlation between the number of visits to the physiotherapist and self-efficacy. The patients with a higher belief in themselves to perform an exercise programme despite different barriers had more visits to the physiotherapist. Gatchel et al. state that high self-efficacy is beneficial when people are confronted with acute or chronic pain and that people with high self-efficacy are more likely to engage in health-promoting behaviours [15]. The sense of self-efficacy can play a large role in how a person deals with challenges [21]. It is possible that physiotherapists spend more time helping patients who are more likely to manage their own exercise programme than the ones in need of more support at the clinic. No other correlation between the number of visits to the physiotherapist and fear of movement, empowerment, or enablement was statistically significant.

The mean number of visits for all diagnosis together was 6.6 (7.9); the median number of visits was 2 with a range between 1 and 28 (Table 2). The results of this current study were similar to research done by Fritz et al. who showed a mean of visits to 6.8 and 63.9% experienced an improved outcome [11].

### Implications for future research

The current study was a pilot and feasibility-study. A full-scale study needs a larger sample size, of 200 patients [43]. To avoid bias, a full-scale study could include other centres [43]. In the process of conducting this feasibility study, we identified variables such as the previous experience of physiotherapy, physical activity, sedentary, self-reported health, and visits to physiotherapists individually and in groups as variables to collect in a future large-scale study. Clearer instructions and a reminder could have improved the answer rates. Future research could focus on the effects of group interventions on results of treatment, the number of visits, and the economy of the health care centre. If there is a relation between self-efficacy, fear of movement, empowerment, or enablement, and the number of visits to the physiotherapist for patients with musculoskeletal disorders, it could be possible to early detect patients at risk of requiring more visits to the physiotherapist and provide early interventions to these factors and patients.

## Conclusion

The results of the study showed that the design is feasible in terms of recruitment of participants and use of questionnaires. In further studies attention needs to be focused on the improvement of the number of completed questionnaires, adding more variables to analyse, and having a much larger study sample. The results of this study indicate that the care of patients with a low level of self-efficacy can be improved.

## Data Availability

The datasets used and analysed during the current study are available from the corresponding author on reasonable request.

## Abbreviations

ESES: Exercise Self-Efficacy Scale
ESES-S: Swedish version of Exercise Self-Efficacy Scale
IQR: Interquartile range
PEI: Patient Enablement Instrument
SD: Standard deviation
TSK: Tampa Scale for Kinesiophobia
TSK-SV: Swedish version of Tampa Scale for Kinesiophobia
